# Organ/space infection is a common cause of high output stoma and outlet obstruction in diverting ileostomy

**DOI:** 10.1186/s12893-020-00734-7

**Published:** 2020-04-28

**Authors:** Yutaro Hara, Takuya Miura, Yoshiyuki Sakamoto, Hajime Morohashi, Hayato Nagase, Kenichi Hakamada

**Affiliations:** Department of Gastroenterological Surgery, Hirosaki University Graduate School of Medicine, Zaifu-cho 5, Hirosaki, 036-8562 Japan

**Keywords:** Diverting ileostomy, High output stoma, Outlet obstruction, Organ/space infection

## Abstract

**Background:**

The objectives of this study are to identify causes of high-output stoma (HOS) and outlet obstruction (OO), which are major complications of diverting ileostomy.

**Methods:**

A retrospective analysis was performed in 103 patients who underwent colorectal surgery and diverting ileostomy between December 2015 and November 2018.

**Results:**

HOS was found in 32 patients (31.1%) and OO in 19 (18.4%). Organ/space surgical site infection (SSI), anastomotic leakage and OO were significant HOS-related factors in univariate analysis, and OO (odds ratio [OR] 3.39, *p* = 0.034) was a independent HOS-related factor in multivariate analysis. Organ/space SSI and male were significant OO-related factors in univariate analysis, and organ/space SSI (OR 3.77, *p* = 0.018) was a independent OO-related factor in multivariate analysis. The white blood cell (WBC) count on postoperative day (POD) 3 was significantly higher in the HOS group compared to the non-HOS group (9765 vs. 8130 /mL, *p* < 0.05), and the WBC count (9400 vs. 7475 /mL, *p* < 0.05) and C-reactive protein level (6.01 vs. 2.92 mg/L, *p* < 0.05) on POD 6 were significantly higher in the OO group compared to the non-OO group.

**Conclusion:**

Organ/space infection is involved in the common pathology of HOS and OO. Decreased intestinal absorption due to intestinal edema caused by organ/space SSI and relative stenosis at the abdominal wall-penetrating site are major causes of HOS and OO.

## Background

Diverting ileostomy reduces the risk of anastomotic leakage (AL) after surgery for rectal cancer, and use of diverting ileostomy has increased [1–3]. However, complications of ileostomy-related high output stoma (HOS) and outlet obstruction (OO) have incidences of 16–23% [4, 5] and 5.6–25.8% [6, 7], respectively. HOS causes dehydration, electrolyte imbalance and renal dysfunction, resulting in a significant decrease of quality of life (QOL). Most studies of HOS have described detection or management, but few have examined the pathology of HOS. Causes of HOS include diabetes, total proctocolectomy, intraabdominal abscess, paralytic ileus, AL and OO, but no clear pathology has been shown [4, 8–12]. OO is defined as intestinal obstruction in an abdominal wall-penetrating site, but differs from general intestinal obstruction because symptoms are relieved by tube insertion from the stoma. Causes of OO include total proctocolectomy and the thickness of the rectus abdominis muscle, but as for HOS, the pathology remains to be elucidated [13–15]. Furthermore, no study has examined HOS and OO simultaneously and the relationship between HOS and OO is unknown. Therefore, this study was performed as a retrospective examination of patients with diverting ileostomy to determine the pathology and relationship of HOS and OO, and to identify related factors.

## Methods

The subjects were 103 consecutive patients who underwent colorectal surgery and diverting ileostomy between December 2015 and November 2018. The study was performed as a retrospective analysis. The indications for diverting ileostomy creation were intersphincteric resection (ISR), preoperative therapy, or male patients with anastomosis just above anal canal after total mesorectal excison. Patients who underwent total proctocolectomy or emergency surgery were often considered for diverting ileostomy creation. The diverting ileostomy site was 40 cm distant from the terminal ileum in the right lower abdomen to penetrate the abdominal wall in the direction to allow lifting of the wall naturally. The aponeurosis of the rectus abdominis muscle was longitudinally incised with a two-finger width. A standardized technique was used to create the loop ileostomy in the all patients.

Patient characteristics of age, sex, disease, body mass index (BMI), diabetes, smoking history, preoperative blood albumin (Alb), preoperative estimated glemerular filtration rate (eGFR) and thickness of the rectus abdominis muscle were examined. The thickness of the rectus abdominis muscle was measured using a slice at the umbilical level on computed tomography (CT) recorded immediately before surgery. A straight line was drawn orthogonally to the horizontal axis at the maximal thickness, and the thickness of the rectus abdominis muscle was determined [14]. Surgical factors, such as operative procedure, approach, lateral lymph node dissection (LLND), operative time, blood loss volume, transfusion, intraoperative fluid, anastomotic procedure were also examined. The preoperative conditions including perforation, stenosis and preoperative chemotherapy were also examined.

Postoperative complications were analyzed using the Clavien-Dindo classification. In our institution, anastomotic infectious complications are divided into AL and organ/space surgical site infection (SSI). AL was defined as clinical symptoms such as fever, abdominal pain and peritoneal irritation, and based on pus-like or stool-like output draining from the pelvic floor, anastomotic dehiscence found in a digital rectal examination, extravasation of endoluminally administered water-soluble contrast enema, and fluid or gas retention surrounding the anastomotic site detected by CT. Organ/space SSI was defined clinical symptoms of intraperitoneal infection without no evidence of AL. It needs only antibiotic therapy for fever, abdominal pain and peritoneal irritation without surgical treatment.

HOS was defined as two-days continuous output of > 1500 mL per day [16]. OO was defined as symptoms of intestinal obstruction, imaging of caliber changes in the abdominal wall-penetrating site in ileostomy by CT, and a condition that was improved by tube retention in the oral stoma [15, 17]. These symptoms and signs were used to confirm the diagnosis of OO. Associations of clinical factors with HOS and OO were examined by Fisher chi-square test and Mann-Whitney U test. Factors with a significant difference (*p* < 0.05) were then evaluated by multivariate analysis. All statistical analyses were conducted using EZR [18].

## Results

### Background of subjects

The median age of the 103 patients was 66 years-old and the median BMI was 22.9 (16.9–38.9) kg/m^2^. Eighty two (80%) patients were male, 16 (15.5%) patients had diabetes, 25 (24.3%) were smokers, and 6 (5.8%) were being treated with steroids. Preoperatively, the Alb level was 4.2 (2.4–5.0) g/dL, eGFR was 74.9 (20.1–135.2) mL/min/1.73 m^2^, and the thickness of the rectus abdominis muscle was 10.25 (5.46–19.14) mm. The underlying diseases were malignant tumor in 84 (81.2%) patients, inflammatory bowel disease in 17 (16.5%), and perforation of colon in 2 (1.9%).

Surgical procedures were low anterior resection in 52 (50.5%) patients, intersphincteric resection in 31 (30.1%), total proctcolectomy in 17 (16.5%), and high anterior resection, sigmoidectomy and ileocecal resection in one subject each. Anastomotic procedures were a double stapling technique in 57 (55.3%) patients and hand-sewn anastomosis in 46 (44.7%). Approaches for intraperitoneal cavity used laparoscopy in 78 (75.7%) patients, a robot-assisted method in 15 (14.6%), and laparotomy in 10 (9.7%). LLND was performed in 28 (27.2%) patients and preoperative chemotherapy in 34 (33%). The median operative time was 319 (123–639) min, median blood loss volume was 60 (0–3550) mL, median intraoperative fluid volume was 2800 (419–8800) mL, and intraoperative transfusion was performed in 10 patients (9.7%). The postoperative complications were AL in 18 (17.5%) patients, organ/space SSI in 39 (37.9%), HOS in 32 (31.1%), and OO in 19 (18.4%). Grade IIIb and IV complications were found in 9 patients (8.7%), of whom 7 had AL (Table 1).

**Table 1:** Clinicopathological characteristics of 103 patients

Variable	Value
Age (year)*	66 (17–82)
Gender, n (%)	
Male	82 (80)
Female	21 (20)
Body mass index (kg/m^2^)*	22.9 (16.9–38.9)
Diabetes, n (%)	16 (15.5)
Smoker, n (%)	25 (24.3)
Steroid user, n (%)	6 (5.8)
Alb (g/dL)*	4.2 (2.4–5)
eGFR (ml/min/1.73m^2^)*	74.9 (20.1–135.2)
Diameter of muscle (mm)	10.25 (5.46–19.14)
Perforation, n (%)	2 (1.9)
Stenosis, n (%)	9 (8.7)
Preoperative chemotherapy, n (%)	34 (33)
Cause of resection, n (%)	
Neoplasia	84 (81.2)
Inflammatory bowel disease	17 (16.5)
Benign pathologies	2 (1.9)
Type of resection, n (%)	
Low anterior resection	52 (50.5)
Intersphincteric resection	31 (30.1)
Total proctocolectomy	17 (16.5)
Others	3
Approach, n (%)	
Laparoscope-assisted surgery	78 (75.7)
Robotic surgery	15 (14.6)
Open surgery	10 (9.7)
Double stapling technique, n (%)	57 (55.3)
Lateral lymph node dissection, n (%)	28 (27.2)
Operation time (min)*	319 (123–639)
Blood loss (ml)*	60 (0–3550)
Blood transfusion, n (%)	10 (9.7)
Replacement fluid volume in the operation (ml)*	2800 (419–8800)
Double stapling technique, n (%)	57 (55.3)
Preoperative chemotherapy, n (%)	34 (33)
Anastomotic leakage, n (%)	18 (17.5)
Organ/space SSI, n (%)	39 (37.9)
High output stoma, n (%)	32 (31.1)
Outlet obstruction, n (%)	19 (18.4)
Complications (Clavien-Dindo), n (%)	
All (I-IV)	69 (67.0)
IIIa	12 (11.7)
IIIb	3 (2.9)
IV	6 (5.8)

*Median (range)

### Analysis of HOS

The median onset time of HOS was postoperative day (POD) 4 (range POD 2–15), the median output volume was 2460 (1800–5450) mL, and the median maximum output volume on the onset day was 3005 (1800–5450) mL (Table 2). Organ/space SSI, AL, and OO were significant HOS-related factors in univariate analysis, and OO (odds ratio [OR] 3.39, *p* = 0.034) remained as a significantly independent factor associated with HOS in multivariate analysis (Table 3). The white blood cell (WBC) count on POD 3 was significantly higher in the HOS group than in the non-HOS group (9765 vs. 8130 /mL, *p* < 0.05) (Table 4). The WBC count on POD 6 and C-reactive protein (CRP) levels on PODs 3 and 6 were also higher in the HOS group.

**Table 2:** Output volume about OO and HOS

	OO	HOS	non-OO&HOS
Onset POD of HOS or OO median (range)	4 (1–14)	4 (2–15)	–
Output volume with onset day (ml) median (range)	1100 (25–3600)	2460 (1800–5450)	–
Maximum volume of stoma output (ml) median (range)	2275 (80–4700)	3005 (1800–5450)	1030 (390–3300)

*HOS* high output stoma, *OO* outlet obstruction, *POD* post operative day

**Table 3:** Univariate and multivariate analyses of clinicopathological variables on High output stoma

Variables		N	Univariate analysis		Multivariate analysis	
			n (%)	*P* value	Odds ratio (95% CI)	*P* value
Age	≥65	50	17 (34.0)			
	< 65	53	15 (28.3)	0.670		
Gender	Female	21	3 (14.3)			
	Male	82	29 (35.4)	0.070		
BMI (kg/m^2^)	< 25	25	9 (36)			
	≥25	78	23 (29.5)	0.621		
Diabetes	No	87	26 (29.9)			
	Yes	16	6 (37.5)	0.565		
Smoker	No	78	22 (28.2)			
	Yes	25	10 (40)	0.323		
Steroid user	No	97	28 (28.9)			
	Yes	6	4 (66.7)	0.073		
Alb	≥4.2	44	14 (31.8)			
	< 4.2	59	18 (30.5)	1		
eGFR	≥60	91	30 (33.0)			
	< 60	12	2 (16.7)	0.333		
Diameter of muscle (mm)	≥10.25	42	12 (28.6)			
	< 10.25	61	20 (32.8)	0.672		
Neoplasia	No	19	9 (47.4)			
	Yes	84	23 (27.4)	0.105		
Perforation	No	101	31 (30.7)			
	Yes	2	1 (50)	0.527		
Stenosis	No	94	28 (29.8)			
	Yes	9	4 (44.4)	0.454		
Operation time (min)	≥319	53	20 (37.7)			
	< 319	50	12 (24)	0.143		
Blood loss (ml)	≥60	50	19 (38)			
	< 60	53	13 (24.5)	0.201		
Total proctocolectomy	No	86	24 (27.9)			
	Yes	17	8 (47)	0.153		
LLND	No	75	24 (32)			
	Yes	28	8 (28.6)	0.814		
Double stapling technique	No	43	17 (39.5)			
	Yes	60	15 (25)	0.134		
Preoperative chemotherapy	No	69	21 (30.4)			
	Yes	34	11 (32.4)	1		
Blood transfusion	No	93	28 (30.1)			
	Yes	10	4 (40)	0.497		
Replacement fluid volume in the operation (ml)	≥2800	53	21 (39.6)			
	< 2800	50	11 (22)	0.059		
Anastomotic leakage	No	85	22 (25.9)			
	Yes	18	10 (55.6)	0.023	2.25 (0.58–8.74)	0.241
Organ space SSI	No	64	13 (20.3)			
	Yes	39	19 (48.7)	0.004	1.98 (0.63–6.27)	0.245
OO	No	84	21 (25)			
	Yes	19	11 (57.9)	0.011	3.39 (1.10–10.5)	0.034

*BMI* body mass index, *LLND* lateral lymph node dissection, *SSI* surgical site infection

**Table 4:** 1, 3, 6 POD WBC and CRP about HOS and non-HOS

	WBC			CRP (mg/L)		
	HOS	non-HOS	*p*-value	HOS	non-HOS	p-value
1 POD	9670 (8200–11,575)	10,170 (8750–11,568)	0.606	6.39 (4.35–10.30)	6.68 (4.93–8.63)	0.724
3 POD	9765 (8058–13,210)	8130 (6950–100,909)	0.015	13.28 (7.60–19.50)	11.787 (7.20–14.27)	0.224
6 POD	8085 (6907–9605)	7540 (6410–8725)	0.122	5.20 (1.69–10.81)	3.01 (1.70–5.71)	0.208

*HOS* high output stoma, *POD* post operative day, *WBC* white blood cell (3300–8600), *CRP* C-reactive protein (0.00–0.14)

### Analysis of OO

The median onset time of OO was POD 4 (range POD 1–14), the median output volume was 1100 (25–3600) mL, and the median maximum output volume on the onset day was 2275 (80–4700) mL (Table 2). Organ/space SSI and male were significant OO-related factors in univariate analysis, but thickness of the rectus abdominis muscle did not show this relationship. Organ/space SSI (OR 3.77, *P* = 0.018) was a significantly independent factor associated with OO in multivariate analysis (Table 5). The WBC count (9400 vs. 7475 /mL, *p* < 0.05) and CRP level (6.01 vs. 2.92 mg/L, p < 0.05) on POD 6 were significantly higher in the OO group than in the non-OO group (Table 6). The WBC count and CRP level on POD 3 were also higher in the OO group. Out of 19 patients in the OO group, 11 patients had HOS simultaneously. In HOS and OO cases, 8 patients had organ/space SSI (72.7%).

**Table 5:** Univariate and multivariate analyses of clinicopathological variables on outlet obstruction

Variables		N	Univariate analysis		Multivariate analysis	
			n (%)	*P* value	Odds ratio (95% CI)	*P* value
Age	≥65	53	10 (18.9)			
	< 65	50	9 (18)	1		
Gender	Female	21	0 (0)			
	Male	82	19 (23.2)	0.011	NA	
BMI (kg/m^2^)	< 25	25	6 (24)			
	≥25	78	13 (16.7)	0.393		
Diabetes	No	87	15 (17)			
	Yes	16	4 (25)	0.129		
Smoker	No	25	5 (20)			
	Yes	78	14 (18.2)	1		
Steroid user	No	97	18 (18.6)			
	Yes	6	1 (16.7)	0.143		
Alb	≥4.2	44	11 (25)			
	< 4.2	59	8 (13.6)	0.199		
eGFR	≥60	91	15 (16.5)			
	< 60	12	4 (33.3)	0.227		
Diameter of muscle (mm)	≥10.25	42	8 (19.0)			
	< 10.25	61	11 (18)	1		
Neoplasia	No	19	3 (15.8)			
	Yes	84	16 (19.0)	1		
Perforation	No	101	19 (18.9)			
	Yes	2	0 (0)	1		
Stenosis	No	94	16 (17.0)			
	Yes	9	3 (33.3)	0.361		
Total proctocolectomy	No	86	16 (18.6)	1		
	Yes	17	3 (17.6)			
LLND	No	75	14 (18.67)			
	Yes	28	5 (17.9)	1		
Double stapling technique	No	43	11 (25.6)			
	Yes	60	8 (13.3)	0.129		
Preoperative chemotherapy	No	69	13 (18.8)			
	Yes	34	6 (17.6)	1		
Operation time (min)	≥319	53	10 (18.9)			
	< 319	50	9 (18)	1		
Blood loss (ml)	≥60	50	10 (20)			
	< 60	53	9 (17.0)	0.801		
Blood transfusion	No	93	16 (17.2)			
	Yes	10	3 (30)	0.388		
Replacement fluid volume in the operation (ml)	≥2800	53	9 (17.0)			
	< 2800	50	10 (20)	0.801		
Anastomotic leakage	No	85	15 (17.6)			
	Yes	18	4 (22.2)	0.518		
Organ space SSI	No	64	6 (9.4)			
	Yes	39	13 (33.3)	0.004	3.77 (1.26–11.3)	0.018

*BMI* body mass index, *LLND* lateral lymph node dissection, *SSI* surgical site infection

**Table 6:** 1, 3, 6 POD WBC and CRP about OO and non-OO

	WBC			CRP (mg/L)		
	OO	non-OO	p-value	OO	non-OO	p-value
1 POD	10,950 (8835–12,900)	10,060 (8410–11,500)	0.470	5.52 (4.20–7.91)	6.78 (4.92–9.91)	0.508
3 POD	10,180 (7775–12,690)	8130 (7195–10,343)	0.068	14.0 (9.75–22.33)	11.73 (7.27–15.27)	0.099
6 POD	9400 (7050–10,007)	7475 (6408–8810)	0.031	6.01 (2.97–11.05)	2.92 (1.50–6.49)	0.023

*OO* outlet obstruction, *POD* post operative day, *WBC* white blood cell (3300–8600), *CRP* C-reactive protein (0.00–0.14)

## Discussion

The criteria for the creation of diverting stoma vary among institutions. A meta-analysis of the significance of diverting stoma in rectal cancer showed that the anastomosis close to the anus was protected by diverting stoma [19]. A multicenter study in Japan showed that diverting stoma did not decrease the incidence of AL, but reduced the severity [1], and three quarters of patients with AL avoided reoperation, showing the usefulness of diverting stoma. In addition, a multicenter study confirmed that oncological safety is comparable in sphincter-preserving surgery and abdominoperineal resection of locally advanced lower rectal cancer [20]. Therefore, the diverting stoma will continue to be created in patients with rectal cancer.

Intraabdominal abscess, paralytic ileus, AL and OO have previously been identified as risk factors for HOS [4, 8–12]. Total proctocolectomy and a history of diabetes have also been suggested to be preoperative predictors of HOS [21], but these factors were not identified as significant risk factors in this study. The reported risk factors for OO are total proctocolectomy and thickness of the rectus abdominis muscle at the stoma-penetrating site [13–15]. However, these factors also had no marked relationship with OO in this study. Infection in organ/space site was associated with the causes of HOS and OO. HOS and OO were associated with the same factor which suggested similar pathology.

WBCs and CRP were examined on PODs 1, 3 and 6 as markers that reflect infectious conditions. The HOS and OO groups both had higher WBC counts and CRP levels on PODs 3 and 6 compared to the non-HOS and non-OO groups. The WBC count on POD 1 has previously been suggested to be a predictor of HOS [22], but this relationship was not significant in this study. The high WBC counts and CRP levels on PODs 3 and 6 show a prolonged postoperative infection in organ/space site, and suggest that intestinal edema and a prolonged decrease in intestinal absorption, which may be caused by infection in organ/space site, contribute to the pathology of HOS and OO. Consequently, patients with organ/space SSI should be managed with the probability of HOS and OO kept in mind. The median onset time of HOS and OO was POD 4, but some patients experienced HOS and OO on POD 1 and 2. Therefore, HOS and OO may be useful for an early sign suggesting infection in organ/space site.

In terms of output volume of OO, many patients had output volume > 1000 mL on the day of clinical diagnosis of OO. It may be because of prompt tube insertion in the stoma for patients with symptoms such as abdominal distension. In this study, all subjects who developed OO were fully improved by conservative treatment such as tube insertion in the oral stoma. Therefore, the OO pathology is relative stenosis of an abdominal wall-penetrating site of a stoma due to intestinal edema caused by infection in organ/space site. Thus, OO should be differentiated from general structural intestinal obstruction. And it may better to say relative outlet stenosis.

The limitation of this study is its performance at a single-center study and lack of external validity. There is an possibility that OO was a real structural obstruction leading to HOS. However, the results of the study suggest that infection in organ/space site is the major cause of HOS and OO. Consequently, the most important countermeasure for reducing HOS and OO is to decrease the incidence of AL and infection in organ/space site. Intraoperative assessment of tissue perfusion during colorectal resection using indocyanine green (ICG) [23], insertion of an anal drain to decrease pressure in the anastomosed region [24], and stabilization of procedures using robotic-assisted surgery [25–27] may potentially improve outcomes. It is critical to treat organ/space infection with consideration of the possibility of HOS and OO onset.

## Conclusion

HOS and OO were found in 31 and 18% of subjects who underwent colorectal surgery and diverting ileostomy, respectively. Infection in the organ/space was associated with the causes of HOS and OO. HOS and OO were associated with the same factor which suggested similar pathology.

## Data Availability

The datasets used and/or analyzed during the current study are available from the corresponding author on reasonable request.

## Abbreviations

AL: Anastomotic leakage
HOS: High output stoma
OO: Outlet obstruction
QOL: Quality of life
ISR: Intersphincteric resection
BMI: Body mass index
Alb: Albumin
eGFR: Estimated glemerular filtration rate
CT: Computed tomography
LLND: Lateral lymph node dissection
SSI: Surgical site infection
POD: Postoperative day
OR: Odds ratio
WBC: White blood cell
CRP: C-reactive protein
ICG: Indocyanine green
